# 低剂量螺旋CT肺癌筛查研究进展

**DOI:** 10.3779/j.issn.1009-3419.2020.101.40

**Published:** 2020-10-20

**Authors:** 梦娜 韦, 友林 乔

**Affiliations:** 100021 北京，国家癌症中心/国家肿瘤临床医学研究中心/中国医学科学院北京协和医学院肿瘤医院流行病学室 Department of Cancer Epidemiology, National Cancer Center/National Clinical Research Center for Cancer/Cancer Hospital, Chinese Academy of Medical Sciences and Peking Union Medical College, Beijing 100021, China

**Keywords:** 肺肿瘤, 低剂量螺旋计算机断层扫描, 筛查, Lung neoplasms, Low dose helical computed tomography, Screening

## Abstract

肺癌严重危害人类健康，呈现疾病负担重、晚期肺癌占比大和5年生存率低的现状。因此，开展肺癌人群筛查以提高早诊早治至关重要。美国肺癌筛查试验（National Lung Screening Trial, NLST）公布的低剂量螺旋计算机断层扫描（low dose helical computed tomography, LDCT）筛查可以降低肺癌死亡率，给肺癌的早诊早治带来了希望。近年来，LDCT肺癌筛查研究不断取得新进展。然而，目前关于LDCT用于肺癌筛查是否可以大规模推广应用仍存在争议。本文将从LDCT肺癌筛查的发展史、高危人群选择、结节管理、筛查效果、筛查接受度和成本效益等方面进行综述。

## Abstract (en)

Lung cancer which represents characteristics of a heavy disease burden, a large proportion of advanced lung cancer and a low five-year survival rate is a threat to human health. It is essential to implement population-based lung cancer screening to improve early detection and early treatment. The National Lung Screening Trial (NLST) demonstrated that screening with low dose helical computed tomography (LDCT) may decrease lung cancer mortality, which brings hope for the early diagnosis and treatment of lung cancer. In recent years, great progresses have been made on research of lung cancer screening with LDCT. However, whether LDCT could be applied to large population-based lung cancer screening projects is still under debate. In this paper, we review the recent progresses on history of lung cancer screening with LDCT, selection of high-risk individuals, management of pulmonary nodules, performance of screening, acceptance of LDCT and cost-effectiveness.

肺癌是全球发病和死亡最高的恶性肿瘤，Globacan 2018数据显示，2018年全球新发肺癌病例约210万，死亡病例约180万^[1]^。大多数肺癌患者在诊断时已是晚期，无法接受手术治疗^[2, 3]^。因此，开展肺癌筛查以提高早诊率、治疗率，对改善患者生存、降低肺癌死亡率具有重要意义。

自20世纪50年代起至今，多个机构开展了肺癌筛查研究。研究^[4]^显示：X线和痰液细胞学可以有效检出早期肺癌，改善生存率，但不能降低肺癌死亡率。目前，也有关于肺癌早期诊断生物标志物的研究，但尚未在临床应用和筛查中推广^[5]^。低剂量螺旋计算机断层扫描（low dose helical computed tomography, LDCT）用于肺癌筛查是目前的研究热点之一。本研究将从LDCT肺癌筛查发展史、高危人群选择、结节管理、筛查效果、筛查接受度和成本效益等方面进行综述。

## 1 LDCT肺癌筛查发展史

20世纪90年代，Naidich等^[6]^首次提出LDCT可作为肺癌筛查新方法。早期开展的LDCT肺癌筛查研究多为探索性研究，样本量差异较大，最少的为87人^[7]^，最多的为31, 567人^[8]^。著名的早期肺癌行动计划（Early Lung Cancer Action Program, ELCAP）纳入了1, 000名60岁、吸烟指数 > 10的无症状高危个体接受年度胸部X线筛查和LDCT筛查，研究发现LDCT检出肺癌的灵敏度比胸部X线高，且检出肺癌多为早期癌^[9]^；随后ELCAP扩展为国际合作项目（International Early Lung Cancer Action Program, I-ELCAP），筛查对象多达30, 000人，研究结果和ELCAP一致，且肺癌患者的生存率得到了改善^[8]^。

探索性研究后，全球各国开展了多项LDCT肺癌筛查的随机对照研究，其中美国肺癌筛查试验（National Lung Screening Trial, NLST）是著名的随机对照试验之一。NLST纳入了53, 454名高危个体，随机分为LDCT筛查组和胸部X线筛查组，接受每年一次、共3轮的筛查，结果首次证明了LDCT筛查组与胸部X线筛查组相比，肺癌死亡率下降了20%（RR=0.8; 95%CI: 0.73-0.93）^[10]^。NLST结果公布后，美国开始推荐LDCT用于本国高危人群肺癌筛查^[11]^。其他一些国家和地区也开展了多项LDCT肺癌筛查随机试验研究。目前荷兰-比利时随机肺癌筛查试验（Nederlands-Leuvens Longkanker Screenings Onderzoek, NELSON）^[12]^和德国肺癌筛查干预试验（German Lung cancer Screening Intervention, LUSI）^[13]^也公布了LDCT筛查可以降低肺癌死亡率的结果，欧洲开始考虑推荐LDCT用于肺癌筛查。

中国也开展了不同类型的LDCT肺癌筛查项目，探讨LDCT在中国地区用于肺癌筛查的效果。2009年我国将肺癌纳入了国家医改重大专项“农村癌症早诊早治项目”，启动了我国肺癌高危人群筛查工作^[14]^；2012年启动的“城市癌症早诊早治项目”也将肺癌纳入筛查项目^[15]^；此外，上海交通大学胸科医院在上海开展了LDCT肺癌筛查随机对照试验^[16]^。但目前我国开展的肺癌筛查项目基本只报告了基线筛查数据，缺少长期随访数据。

## 2 LDCT肺癌筛查高危人群选择

目前全球开展的LDCT肺癌筛查研究，高危人群选择主要有两种方式：一种是以年龄、吸烟为主要危险因素，参考或不参考其他危险因素；另一方式是基于风险预测模型进行选择。但不同研究间筛查起止年龄和吸烟史的定义差异较大；不同风险预测模型纳入的预测因子各不相同，模型效果存在差异。

国外开展的LDCT肺癌筛查项目的开始年龄在40岁-60岁间^[8, 17, 18]^，停止筛查的最小年龄为69岁^[19, 20]^，大多数项目停止筛查的年龄为74岁左右^[18]^，而有的项目不限制停止筛查的年龄^[8, 21]^。吸烟是选择筛查人群的另一主要危险因素，大多项目选择重度吸烟者或戒烟者作为目标人群^[20, 21]^；而日本的一项随机对照试验^[22]^的筛查对象为非吸烟或轻度吸烟者。风险预测模型是选择筛查目标人群的另一方法。在国外，常用的高危人群风险预测模型有：利物浦预测模型（Liverpool Lung Project risk model, LLP）^[23]^和加拿大肺癌模型（Pan-Canadian Early Detection of Lung Cancer Study, PanCan）^[24]^等。不同模型纳入的预测因子各有差异，常见的预测因子有年龄、性别、种族、教育水平、身体质量指数、家族史、吸烟史等，有些模型还考虑了性别种族间的交互作用和职业暴露^[23-25]^。

由于国情差异，在借鉴参考国外经验时，应探索符合我国国情的高危人群选择标准。我国也开展了多项LDCT肺癌筛查研究，但高危人群选择标准却各有不同。《中国肺癌低剂量螺旋CT筛查指南》2018版主要依据年龄（50岁-74岁）和吸烟史（≥20包·年），如已经戒烟则戒烟时间不超过5年；同时考虑某些肺癌高发区的特异性危险因素，如宣威地区室内燃煤和个旧地区的职业暴露史^[26]^。上海一个基于社区的前瞻性随机对照试验^[16]^也主要依据年龄（45岁-70岁）和吸烟史（≥20包·年），但戒烟时间的标准更严格，戒烟时间不超过15年的仍然需要筛查，同时也考虑其他危险因素。而另一项在上海开展的多中心前瞻性研究^[27]^选择筛查人群时只考虑了年龄（> 35岁）。2012年启动的城市癌症早诊早治项目中的肺癌筛查则采用高危人群评估模型选择肺癌高危人群^[15]^。

选择合适的目标人群进行LDCT肺癌筛查是实现筛查效益最大化、副作用最小化的关键。不同国家和地区由于肺癌流行特征、风俗人文、自然资源、医疗资源，经济等方面存在差异，在实际开展LDCT肺癌筛查时，应结合实际情况综合考虑，选择最适合筛查的高危人群。有研究^[24, 28, 29]^报道，风险预测模型筛选高危人群的效果优于传统的筛选标准，但现有不同模型间效果差异较大，模型外推的证据依然较少。有些研究者还担心模型太复杂，不利于应用于临床实践，因此开展更多研究来优化现有预测模型、实现更佳的预测效果是将来努力的方向。

## 3 肺结节的管理

LDCT筛查在发现肺部微小病变时，也检出大量良性结节，如NLST中筛查阳性结果有96.4%是假阳性^[10]^，假阳性高不仅增加了医疗负担，还增加了筛查对象的焦虑情绪。因此，如何定义阳性结节是筛查的重要问题之一。

目前，全球关于阳性肺结节定义以及肺结节的管理尚无统一标准。NLST将基线筛查时最大直径≥4 mm的肺结节定义为阳性结节，年度筛查的阳性肺结节定义和基线筛查相同^[18]^；I-ELCAP中阳性结节直径最小阈值比NLST严格，同时参考了结节的密度，将实性/部分实性结节≥5 mm，非实性结节≥8 mm，任何支气管内实性结节定义为筛查阳性；年度筛查则将任何新发结节定义为筛查阳性^[8]^。2018年版《中国肺癌低剂量螺旋CT筛查指南》的基线筛查阳性和随访筛查阳性定义和I-ELCAP具有相似之处，新增气管或/及支气管可疑病变，或LDCT诊断为肺癌的肺部单发、多发结节或肺癌包块为阳性；年度筛查则将新发的非钙化性结节或气道病变，或发现原有的结节增大或实性成分增加，定义为阳性^[26]^。

不同的阳性结节定义导致不同研究间阳性率略有差异，需要随访的比例也不同。如基于I-ELCAP进行的一项回顾性分析发现：当将5 mm作为阳性肺结节阈值时，阳性率为16%；当最大直径阈值分别改为6 mm、7 mm、8 mm和9 mm时，阳性率分别为10.2%、7.1%、5.1%和4.0%，随访工作分别下降了36%、56%、68%和75%，有利于减少医疗资源浪费，但如果阳性结节的阈值太严格，可能会导致某些肺癌病例诊断延误，甚至漏诊^[30]^。

目前，国内外各LDCT肺癌筛查指南通常根据结节的大小或体积、密度等特点提出相应处理意见，如美国国立综合癌症网络（National Comprehensive Cancer Network, NCCN）指南^[31]^，Fleischner学会指南^[32]^和中国LDCT肺癌筛查指南^[26]^。有研究报道新发实性肺结节的肺癌风险比基线结节高，且大小比基线结节小^[33, 34]^，实性成分含量不同的结节的良恶性程度也有差异^[35]^。因此，根据不同结节的良恶性程度进行精准管理，减少不必要的活检，提高筛查效益是我们的目标。据此，国内外开发了多个结节风险预测模型^[36-38]^，但中国模型开发数据全部来源于医院。纳入结节风险预测模型的因子包括两大类别^[38, 39]^：研究对象特征（年龄、性别、吸烟史、肺癌家族史、肺气肿等）和结节特征（大小、位置、数量和边缘特征等），有的模型还纳入了分子标志物^[36]^。

不同预测模型的效果也各有差异。McWilliams等^[38]^验证开发的肺结节预测模型表现出较好的预测效果，曲线下面积（area under curve, AUC） > 0.90；Yang等^[36]^开发的模型，训练集的结节风险预测模型AUC为0.915, 1，验证集的AUC为0.583, 6。这些差异可能与模型纳入的预测因子不同有关。不同国家和地区应该结合当地的资源和人群特征，开发符合当地实际情况的结节风险预测模型，真正地实现不同风险不同管理。

## 4 LDCT肺癌筛查效果

### 4.1 提高早诊率

有研究^[9]^发现，LDCT检出肺癌和早期肺癌的能力比胸部X线强。NLST报告LDCT检出肺癌中，Ⅰ期肺癌占54.8%；胸部X线组，Ⅰ期肺癌占37.9%^[40]^。我国开展的LDCT肺癌筛查项目结果和国外的报道一致。Yang等^[16]^在上海开展的基于社区的随机对照研究报告LDCT筛查组和对照组（常规护理）的肺癌检出率分别为1.5%和0.3%，早期肺癌检出率分别为94.1%和20.0%。早期肺癌的检出为患者提供了治疗的机会，有利于改善患者生存状况，实现降低死亡率的目标。

### 4.2 降低死亡率

筛查的主要目的是降低死亡率。虽然全球开展了多项LDCT肺癌筛查研究，但由于研究设计、样本量不足和随访时间不够长等原因，目前，报告肺癌死亡率降低结果的研究不多。随机对照试验NLST是首个报告LDCT筛查可以降低肺癌死亡的研究^[10]^。基于NLST公布的结果，2013年，美国预防服务特别工作组（U.S. Preventive Services Task Force, USPSTF）推荐对本国肺癌高危人群进行LDCT筛查^[11]^。NELSON最近公布了该项目10年随访结果，结果显示男性和女性的肺癌死亡率均下降，且女性肺癌死亡风险下降大于男性^[12]^。LUSI发布的最新随访结果显示：平均随访8.8年后，虽然在男性人群中，肺癌死亡率下降没有统计学差异（HR=0.94, 95%CI: 0.54-1.61），但LDCT筛查可以降低女性肺癌死亡率（HR=0.31, 95%CI: 0.10-0.96）^[13]^。这些结果可以支持LDCT筛查作为高风险人群的防癌措施之一，但不同国家开展具体的筛查项目时应结合本国国情。

### 4.3 辅助诊断其他疾病

此外，LDCT筛查可以辅助诊断其他疾病，如肺气肿、冠状动脉钙化，使筛查受检者额外获益。谢永生等^[41]^对1, 956名受检者的基线LDCT筛查结果进行了分析，结果显示500例患冠状动脉钙化，121例患肺气肿。NLST在筛查期间发现0.39%的筛查对象患有非胸部肿瘤，包括0.26%的肾癌、0.08%的甲状腺癌和0.05%的肝癌^[42]^。虽然LDCT筛查有助于辅助诊断非肺部疾病，但如果不加选择地对偶然的肺外发现进行额外检查可能会给医疗系统带来沉重的负担，因此在报告筛查额外发现时应该慎重。

### 4.4 戒烟

吸烟是肺癌的主要危险因素，研究发现戒烟可以有效降低肺癌死亡率^[43, 44]^。因此将戒烟和筛查结合起来，可以使筛查对象获益更大。Tanner等^[44]^进行的一项系统综述显示，LDCT筛查本身可能不影响吸烟行为，但阳性筛查结果可能与戒烟行为增加有关。一项基于英国肺癌筛查项目（UK Lung Screen, UKLS）开展的研究发现和对照组相比，干预组戒烟率更高，且需要进一步临床干预的筛查对象与对照组（调整OR=2.29，95%CI：1.62-3.22）或筛查结果阴性者（调整OR=2.43，95%CI：1.54-3.84）相比，更有可能长期戒烟^[45]^。

虽然LDCT对筛查对象的戒烟效果尚不明确，但筛查可以为参加筛查的群众提供一个接受戒烟教育的机会，有利于促使筛查对象戒烟。将来应该开展更多研究，探讨LDCT肺癌筛查对戒烟的影响，以及影响戒烟的可能因素，同时在开展肺癌筛查时宣传戒烟的重要性。

## 5 LDCT肺癌筛查意识和接受度

医护人员及群众对LDCT作为肺癌筛查方法的认知、接受度和依从性在一定程度上会影响筛查方法的实施和效果，因此调查他（她）们对LDCT的认知、接受度和依从性以及相关的影响因素对LDCT肺癌筛查的推广具有重要意义。

Raz等^[46]^调查了美国初级保健医护工作者对肺癌筛查指南的了解程度及对LDCT的认知和推荐使用情况，结果发现47%的调查对象了解USPSTF推荐的LDCT肺癌筛查指南，且97%认为LDCT可以降低高危人群肺癌死亡，虽然初级保健医护工作者对LDCT肺癌筛查指南了解较少，但了解筛查指南的医务人员大部分赞同LDCT可以降低肺癌死亡。Shin等^[47]^调查发现89.1%的专家认为LDCT肺癌筛查利大于弊，且79.2%的专家主动向符合条件的高危对象推荐LDCT筛查；但79.8%的专家担心LDCT筛查的高假阳性，37.2%的专家担心筛查的辐射问题。一项对韩国40岁-74岁男性的调查发现，在了解LDCT肺癌筛查的利弊后，肺癌高风险男性的筛查意愿（60.6%）高于普通男性（49.9%）^[48]^。此外，Veliz等^[49]^发现性取向特殊人群（包括同性恋、双性恋和性取向不确定人群）中肺癌高风险人群的比例高于异性恋人群（21.1% *vs* 11.7%），但过去一年内，特殊性取向人群中符合筛查标准但不参加筛查的比例高于异性恋群体（调整OR=3.31，95%CI：1.38-7.94）。

影响参加或推荐LDCT肺癌筛查的因素有^[46, 50-52]^：性别、年龄、经济水平、家庭成员的癌症史、情感认知（如逃避肺癌相关信息）、交通不便、对LDCT肺癌筛查指南的认识、医疗保险的覆盖范围、社会成本、患者的健康优先选择和需要、筛查质量等。因此，通过实施有针对性的干预措施提高相关人员对肺癌的重视程度，对LDCT筛查利弊的正确认识，同时制定科学合理的筛查方案，为筛查提供足够资源，相信可以有效提高LDCT肺癌筛查的依从性和参与率。此外，还需要重视特殊群体的健康需求。

## 6 LDCT肺癌筛查的成本效益

由于资源有限，将LDCT应用于肺癌筛查时，除考虑安全性和有效性外，还需要进行卫生经济学评价。目前关于LDCT肺癌筛查的卫生经济学研究尚存争议，大多数研究基于模型开展，且研究多来自发达国家。

Black等^[53]^从社会角度对NLST进行了卫生经济学评价，结果显示与没有筛查相比，LDCT肺癌筛查增加1个质量调整生命年（quality adjusted life year, QALY）的增量成本效益比（incremental cost-effectiveness ratio, ICER）为81, 000美元，低于美国预先设定的增加一个QALA的阈值（1 QALA/100, 000美元），提示NLST在高危人群中开展LDCT肺癌筛查是经济有效的。Jaine等^[54]^的研究结果却和NLST相反，增加1个QALY的ICER为104, 000美元，高于设定的GDP阈值（30, 000美元），提示在新西兰地区，对该研究定义的目标人群进行LDCT肺癌筛查不具成本效益，按性别、年龄、种族和吸烟状况进行亚组分析后依然不具成本效益。

中国LDCT肺癌筛查成本效益的研究较少。一项对中国台湾吸烟高危人群进行的研究^[55]^显示：增加1个QALA的ICER为19, 683美元，与2013年台湾GDP（20, 925美元）比较，在台湾地区对高危吸烟者实施肺癌LDCT筛查经济有效。中国大陆的卫生经济学研究尚处于初级阶段，尚无进行增量成本分析，研究人群也多为健康体检人群^[56, 57]^。刘成成等^[58]^对全球肺癌筛查卫生经济学研究进行了系统评价，结果初步提示在发达地区对高危人群实施LDCT肺癌筛查经济有效，可以为证据有限的欠发达地区提供参考。

在评价LDCT肺癌筛查的成本效益时容易受到以下因素影响^[54, 59-61]^：筛查人群的戒烟率、诊断时的肺癌分期、卫生系统成本、预期降低的死亡率、筛查价格、筛查方案、筛查参与情况、筛查方法的灵敏度和特异度等。因此，不同国家和地区在评估LDCT肺癌筛查的成本效益或参考现有文献进行评估时，应考虑上述因素在本地的实际情况。

## 7 LDCT肺癌筛查的其他问题

目前，开展LDCT肺癌筛查还存在一些需要解决的其他问题。如过度诊断问题。LDCT可以有效检出早期肺癌，但可能也会检出一些惰性肿瘤。NLST中LDCT检出肺癌有超过18%为惰性肿瘤^[62]^；Brodersen等^[63]^最近发表的对LDCT肺癌筛查随机试验进行的*meta*分析发现：可能有49%筛查发现的肿瘤属于过度诊断。过度诊断会导致不必要的治疗，不仅增加了患者经济负担，还会引起心理问题，因此在实施大规模LDCT肺癌筛查前应考虑过度诊断问题。另一问题是最佳筛查间隔。目前开展项目的筛查间隔多为1年^[13]^，NELSON试验LDCT筛查时间则为第1年、第2年和第4年，最佳筛查间隔目前尚无确切定论^[64]^。一项对NLST进行的回顾性分析^[65]^发现基线LDCT肺癌筛查结果阴性者的肺癌发病比参加基线筛查的所有人低，提示筛查结果阴性的筛查对象可以适当延长筛查间隔，应针对具体的筛查结果实行精准筛查，但需要更多研究来探索可行性。虽然近年来也有探索肺癌筛查早期诊断标志物的研究，某些标志物在肺癌的检出方面也表现出一定潜力，但多处于科研阶段，尚未用于临床或人群筛查。此外，参加筛查可能会给筛查对象带来焦虑、紧张情绪，尤其是筛查结果阳性的筛查对象^[66]^。因此，在开展LDCT肺癌筛查的时候，除关注筛查带来的躯体健康，还需要重视筛查对象的精神健康。

## 8 总结和展望

LDCT筛查可以降低肺癌死亡率，对于实现肺癌的早诊早治具有重要意义。多个国家和地区开展了多项研究对高危人群实施LDCT肺癌筛查，虽然LDCT用于肺癌筛查还存在一些问题需要解决，但随着筛查方案的不断完善，分子标志物研究的日趋成熟以及人工智能在肺癌筛查诊断方面的不断发展，相信肺癌的早诊早治工作将迎来新的希望。我国肺癌疾病负担重，但LDCT肺癌筛查研究仍处于初级阶段，筛查方案大多基于国外的筛查经验制定，且缺乏大样本、长期随访数据，因此有必要开展更多研究，尽快建立符合中国具体国情又具有良好卫生经济学效益的肺癌筛查方案。
